# Optimal Design of Alkaline–Surfactant–Polymer Flooding under Low Salinity Environment

**DOI:** 10.3390/polym12030626

**Published:** 2020-03-09

**Authors:** Adi Novriansyah, Wisup Bae, Changhyup Park, Asep K. Permadi, Shabrina Sri Riswati

**Affiliations:** 1 Department of Energy and Mineral Resources Engineering, Sejong University, Seoul 05006, Korea; anba11181@gmail.com (A.N.); shabrina.riswati@gmail.com (S.S.R.); 2 Department of Energy and Resources Engineering, Kangwon National University, Chuncheon, Kangwon 24341, Korea; 3 Petroleum Engineering Department, Bandung Institute of Technology, Bandung 40132, Indonesia; asepkpermadi@tm.itb.ac.id

**Keywords:** low salinity, alkaline–surfactant–polymer flooding, optimal design, optimal salinity, co-solvent

## Abstract

This paper presents an optimal design of alkaline–surfactant–polymer (ASP) flooding and an experimental analysis on the effects of ASP components under low formation salinity, where the assignment of salinity gradients and various phase types are limited. The phase behavior and coreflooding tests confirmed the ASP formula is optimal, i.e., 1 wt % sodium carbonate (Na_2_CO_3_) as the alkaline, 1:4 weight ratio for linear alkylbenzene sulfonate (LAS) and dioctyl sulfosuccinate (DOSS) as a surfactant, 5 wt % diethylene glycol monobutyl ether (DGBE) as a co-solvent, and hydrolyzed polyacrylamide (HPAM) as a polymer. The salinity scan was used to determine that the optimum salinity was around 1.25 wt % NaCl and its solubilization ratio was favorable, i.e., approximately 21 mL/mL. The filtration ratio determines the polymer concentrations, i.e., 3000 or 3300 mg/L, with a reduced risk of plugging through pore throats. The coreflooding test confirmed the field applicability of the proposed ASP formula with an 86.2% recovery rate of residual oil after extensive waterflooding. The optimal design for ASP flooding successfully generated phase types through the modification of salinity and can be applicable to the low-salinity environment.

## 1. Introduction

Alkaline–surfactant–polymer (ASP) flooding intends to integrate a synergy of chemical mixtures, e.g., alkalis, surfactants, co-solvents, and polymers, in order to recover residual oil [1,2,3,4,5]: alkalis and surfactants mobilize residual oil and reduce the interfacial tension between the displacing phase and the oil; polymer slug enhances mobility ratios and volumetric sweep efficiencies. Successful ASP flooding can be achieved by designing an optimal ASP formula that is closely related to phase types, i.e., microemulsion, such as Winsor types I (II-), II (II+), and III, which formation salinity influences significantly. The design defines the optimum salinity showing the equal solubilization of oil and water and determines the amounts and types of chemical mixtures that can be used to achieve the desired performances, e.g., to adequately reduce the interfacial tension. The lowest interfacial tension and the middle-phase microemulsions, i.e., Winsor type III, are available at a near-optimum salinity, so that it has been a key guideline in the design of ASP flooding.

A low-salinity environment, i.e., the salinity of formation brine is below 1 wt % NaCl [6], causes the challenge of generating various phase (microemulsion) types and salinity gradients. Generally, the optimum salinity is higher than the formation salinity in low-salinity conditions, and thereby the phase type created by ASP slug would remain as Winsor type I, which has a higher interfacial tension. Occurrence of low electrolyte conditions is helpful in the surfactant solubilization process within the aqueous phase. Low electrolyte conditions and co-solvent addition may lower the potential of forming gels, liquid crystals, macroemulsions, and arrival of equilibrium conditions due to the existence of the salting-in phenomenon [7]. As a result of this limited phase type of displacing the ASP mixture, it fails to mobilize the residual oil in such a way that the performances would be poor.

Another challenge of the low-salinity environment is the inability to set varying salinities based on ASP components; the high value is better for preflush, the low salinity could be positive on a polymer drive by maintaining a consistent polymer viscosity, while salinity close to the optimum salinity helps mobilize residual oil through ASP slug. The salinity gradient, i.e., the modification of salinities from the preflush, ASP mixture, and polymer in sequence, has been studied in an effective way to produce more residual oil [2,8,9,10,11,12,13]. Difficulty in maintaining designed salinities at specific locations and conditions inhibits the positive effects of the salinity gradient, e.g., the mixture of formation brine, and ASP slug can change salinities during fluid transport processes. Sheng [8] proposed salinity profiles while placing two salinity guards between surfactant and polymer slug followed by drive slug with salinity lesser than the lower bound of a Winsor type III microemulsion. Levitt et al. [9] investigated the effect of the nonsalinity gradient at a low-saline reservoir and suggested that the salinity increment of ASP slug might avoid salinity loss through dispersion. Gregersen et al. [10] analyzed the impacts of anhydrite on ASP performance under low-salinity conditions. Battistutta et al. [11] analyzed ASP flooding under optimum salinity conditions, i.e., 2.5–4.5 wt % NaCl, and showed the alteration of ASP slug would be negligible at this high salinity. Chen et al. [12] performed ASP coreflooding experiments using different III–I, I–III–I, II–III–I, and III–III–III salinity profiles and concluded that negative salinity was favorable. Riswati et al. [13] examined the design of ASP phase types by changing salinity gradients and showed that Winsor type II–III–I salinity gradients were more likely to be successful in the recovery of more light crude oil. In lab-scaled coreflooding tests, high-salinity preflushes may generate type II salinity profiles favorable for alkalis, although field applications are limited under low formation salinity. In a low-saline reservoir, formation brine can govern the entire ASP flooding, and thereby the salinity adjustment through the preflush waterflooding is not applicable. Additional costs are also required to prepare high salinity.

The aim of this research was to analyze an optimal design of ASP flooding under the low-salinity environment and examine the field applicability with preflushing and polymer-drive processes using in situ brine, i.e., low-saline brine. Although extensive waterflooding was carried out to prevent the recovery of residual oil, the possibility to recover additional oil was discussed.

## 2. Methodology

### 2.1. Properties of Formation Brine and Crude Oil

Crude oil was sampled from an onshore sandstone reservoir of the South Sumatra Basin, Indonesia. The pay zone thickness was approximately 3 m, from 630 to 633 m true vertical depth (TVD). The formation brine had approximately 6560 mg/L (part per million (ppm)) total dissolved solids (TDS). The pH of brine was 7.99 at 25 °C. Table 1 presents a summary of the components of the crude oil sampled. The oil gravity was about 25° API (The American Petroleum Institute gravity; 0.903 g/cm^3^), the viscosity was 0.6 centipoise at 60°, and the acid number was 0.014 mg KOH/g oil, so this sample was categorized as a medium-gravity sample with a low acid number. The crude oil contained 11 mol % of methane (CH_4_), 13 mol % of hexane (C_6_H_14_), and 68 mol % of a heavy component (C_7_H_16_^+^). There was no presence of hydrogen sulfide (H_2_S).

### 2.2. Experimental Procedure

The experiments were divided into the following tests: the phase behavior test to find the best-performing formulation of the ASP elements, the filtration ratio (FR) test to examine the polymer transport stability, and the coreflooding test to estimate the actual flow performances of the ASP formula determined. The alkali was selected to be sodium carbonate (Na_2_CO_3_; Daejung Chemicals, Goryeong, Korea), of which its effectiveness has been validated [4,14,15]. The polymer was hydrolyzed polyacrylamide (HPAM), Alcoflood 955 polymer with a molecular weight around 4.5 million Dalton (Ciba Specialty, Basel, Switzerland). Sodium chloride (NaCl; Daejung Chemicals, Goryeong, Korea) was utilized to make synthetic brine. Two anionic surfactants, i.e., linear alkylbenzene sulfonate (LAS) and C8–C8 double-tail dioctyl sulfosuccinate (DOSS) (Akyung Chemical, Seoul, Korea), were utilized in this study. LAS is a clear liquid surfactant with good thermal stability and is extensively used as a household detergent, while DOSS has been commonly used for an oil spill dispersant [16,17]. The LAS-DOSS combination is effective in low-salinity reservoirs, because the existence of DOSS can be expected to decrease the optimum salinity of LAS, i.e., the optimum salinity of the surfactant mixture becomes lower. Isobutyl alcohol (IBA; Sigma-Aldrich, St. Louis, MO, USA) and diethylene glycol monobutyl ether (DGBE; Daejung Chemicals, Goryeong, Korea) were prepared as co-solvents. DGBE has the longer carbon chain than IBA. Both solvents have similar densities, that is, 0.955 g/cm^3^ for DGBE and 0.803 g/cm^3^ for IBA. The melting and boiling temperatures of IBA are lower than those of DGBE. The flashing temperature of DGBE is 100 °C, and the flashing temperature of IBA is 73 °C, which is lower than that of DGBE. Moreover, 8.7 g of IBA was capable to dissolve in 100 g water, and DGBE is a soluble substance in water, ethanol, ethyl ether, and acetone. Table 2 and Table 3 summarize the properties of available surfactants and co-solvents (LAS and DOSS were prepared as surfactants, and IBA and DGBE were prepared as co-solvents).

#### 2.2.1. Phase Behavior Test

By using the phase behavior test, phase types with salinity changes were observed, and the optimum salinities at the same solubilization ratio for oil and water were determined. It utilized serial 5 mL with 0.1 mL markings silicate pipettes (Witeg Diffico, Wertheim, Germany). All the aqueous solutions and crude oil were added precisely into pipettes using an electric pipette dispenser (Eppendorf International, Hauppauge, NY, USA). The salinity was varied by diluting NaCl with deionized water from Direct-Q Millipore (Youngin, Seoul, Korea). Brine was then added into a pipette with a 1:1 (weight ratio; *wt*/*wt*) aqueous phase (alkali and surfactant mixture)-to-crude oil ratio. The top of the glass was sealed with silicon grease to separate water from the volatile component inside the pipette. All pipettes were shaken up for about 2 min and arranged in a pipe rack in ascending order of brine salinity. The volumes of water, oil, and microemulsion phases were measured at the equilibrium condition, when there was no significant alteration of phase volume observed. Equation (1) was used to calculate the solubilization ratios for oil and water, as shown below:

(1) $$\{\begin{array}{c} \sigma_{o}=\frac{V_{o}}{V_{s}}\\ \sigma_{w}=\frac{V_{w}}{V_{s}} \end{array}\;,$$

where σ (mL/mL) represents the solubilization ratio and V  (mL) is the phase volume within the microemulsion at the equilibrium; the subscripts *o*, *w*, and *s* denote the phases of the oil, water, and surfactant, respectively. Equation (2) was used to estimate the interfacial tension at the optimum salinity [18]:

(2) $$\gamma =\frac{0.3}{{\left(\sigma_{opt}\right)}^{2}\;}\;,$$

where *σ* represents the interfacial tension (mN/m) and $\sigma_{opt}$ is the water or oil solubilization ratio determined at the optimum salinity. The criterion of the solubilization ratio at the optimum salinity is higher or equal to 10 mL/mL, in order to accomplish the desired ultralow interfacial tension, i.e., when the oil solubilization ratio is 10 mL/mL, the interfacial tension is 0.003 mN/m [2,13,19].

#### 2.2.2. FR Test

The FR reflects whether the polymer solution is free of aggregates, which yields a plugging phenomenon at the pore throats. Two different concentrations of the polymer, i.e., 3000 and 3300 mg/L, were assessed. The experimental apparatus consisted of a 2 μm filter membrane representing a porous medium, a filtration chamber, and a volumetric flask, which acted as an effluent collector. Both polymer solutions were prepared using 200 mL each. The polymer was poured into the filtration chamber equipped, which had the filter membrane. The filtration chamber was connected to a nitrogen bottle with a tubing line. Nitrogen was utilized to displace the polymer inside the filtration chamber and establish flow through the filter membrane.

The experiments were carried out under a constant pressure, 25 psi (lb/in^2^). The volumetric flask collected the effluent volume from the chamber, and the time was recorded for every 20 mL incremental of the effluent volume. Nitrogen was injected continuously, until all samples were completely drained from the filtration chamber. Deionized water was used to flush the filtration chamber and thus changed the polymer concentrations. The FR was written as:

(3) $$FR=\frac{t_{200\;\mathrm{mL}}-t_{180\;\mathrm{mL}}}{t_{80\;\mathrm{mL}}-t_{60\;\mathrm{mL}}}$$

where *t* denotes the time recorded at a specific effluent volume and the subscripts 100, 200, and 300 mL represent the effluent volumes. A FR value less than 1.2 is commonly acceptable for nonoccurrence of polymer hydration [20].

#### 2.2.3. Coreflooding Test 

**Figure 1:**
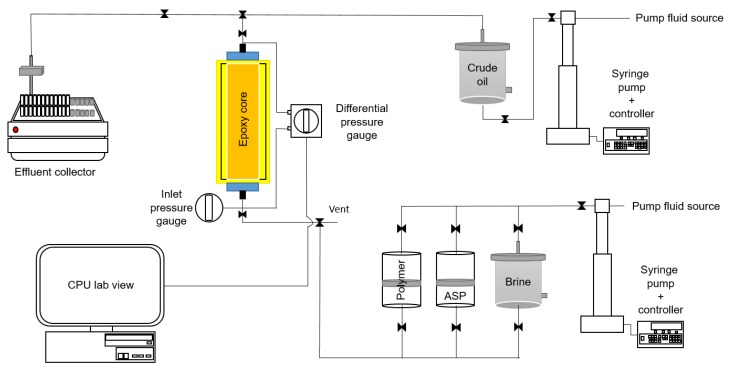
Experimental apparatus for coreflooding tests.

Table 4 shows the properties of two cylindrical Berea sandstone cores used in the coreflooding test. Both cores had similar rock properties, e.g., the average absolute permeability ranges from 180 to 200 millidarcy (md). They were placed in an epoxy coreflooding system, since the adopted pressure was relatively below 100 psi (Figure 1). In the epoxy system, both ends of the rock samples were fixed with sealing tube-fitting caps. Each cap was glued with a quick drying epoxy paste and left to dry for 30 min, until the caps adhered tightly to the ends of core samples. An aluminum foil with silicone sealant covered the surface of the rock sample to prevent direct contact between the epoxy and the core surface. The core was placed inside a polycarbonate cylinder, where one end was glued on a paper sheet with silicon to avoid leakage during the pouring process. Once the silicone was hardened, a low-set epoxy comprised of a 2:1 (*wt/wt*) resin-to-hardener ratio was poured into an annulus between the core and the polycarbonate cylinder. The epoxy covered all rock surfaces to ensure that no air was trapped inside the epoxy. This epoxy core holder system was left to dry at 20 °C for a day to harden the epoxy. Two valves, used to control the fluids, were attached at both ends of the epoxy core after hardening the epoxy.

Two Teledyne ISCO syringe pumps (Lincoln, NE, USA) were used to inject aqueous fluids and crude oil into the epoxy core system. As shown in Figure 1, water was injected as a driving fluid into a crude oil chamber. The driving fluid pushed crude oil inside the chamber towards the epoxy core system. Crude oil was injected into the epoxy core to set up the initial reservoir condition. Before starting the tests, a vacuum pump was used to evacuate any trapped air within epoxy cores. An absolute-pressure gauge was used to measure the inlet pressure, and a differential-pressure transducer was adopted to determine the pressure difference between the inlet and the outlet.

Extensive waterflooding was carried out using the formation brine (0.6 wt % NaCl), until no oil was recovered. Brine saturated the epoxy core system, and then crude oil was injected into the rock core at a constant pressure of 80 psi from the top to the bottom of the vertical epoxy core. The reason for the downward injection is that the differential density between brine and crude oil was able to accelerate the oil flow rather than the opposite case. The oil injection continued, until no brine was observed at the outlet, and then the rock core was aged inside an oven at 80 °C for 72 h to alter the wettability from water-wet to mixed-wet conditions. After the completion of the aging process, formation brine was injected continuously into the core as a preflush fluid. The injection volumetric velocity was maintained at 0.24 cm^3^/min (approximately 1 ft/day superficial velocity), until no oil was recovered [2]. The oil cut and residual oil recovery were observed. Oil cut was defined as the ratio of a produced oil volume to a total fluid production, while the cumulative residual oil recovery ($f_{op}$) was defined as Equation (4):

(4) $$f_{op}=\frac{\sum V_{oi}}{V_{orw}},$$

where $V_{oi}$ (mL) is the produced oil volume in the tube and $V_{orw}$ (mL) represents the remaining oil volume after the waterflooding.

## 3. Results and Discussion

### 3.1. Optimal ASP Formulation and Polymer Concentration

**Figure 2:**
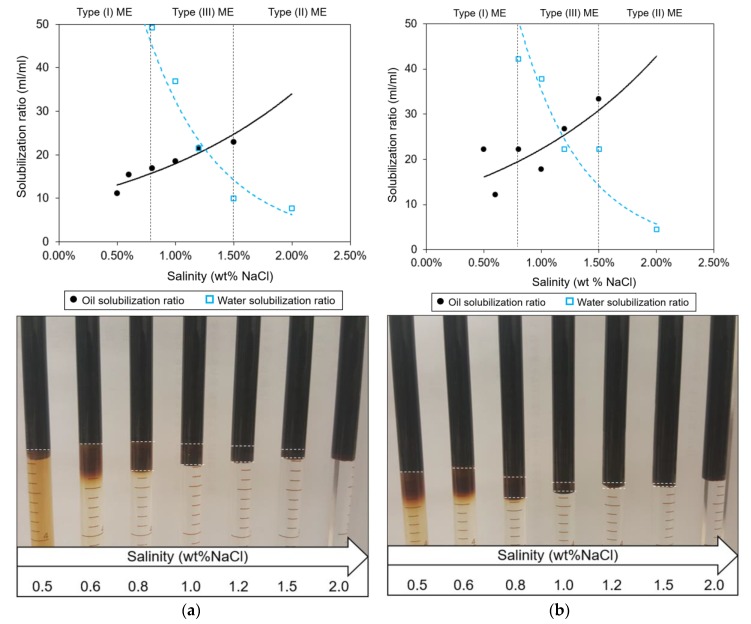
Plot of solubilization ratio with increasing salinities to determine the optimum salinity and phase type with two formulas: (**a**) 1 wt % Na_2_CO_3_ as the alkali, 5 wt % DGBE as the co-solvent, and 1 wt % 1:4 (*wt/wt*) LAS:DOSS mixture as the surfactant (PB5); and (**b**) 1 wt % Na_2_CO_3_ as the alkali, 5 wt % DGBE as the co-solvent, and 0.9 wt % 1:4 (*wt/wt*) LAS:DOSS mixture as the surfactant (PB6). The horizontal dashed lines in the lower-side pictures represent the boundaries of a microemulsion. The weight percentage range of NaCl was assumed to be from 0.8 to 1.5 wt % in the experiment.

Two formulas, i.e., PB5 and PB6, showed the optimum solubilization ratio over 10 mL/mL, so that they were examined as the candidates of coreflooding tests (Table 5). Figure 2 depicts the changes of oil and water solubilization ratios and aqueous salinities for PB5 and PB6. The difference between two cases was negligible; a 0.1 wt % surfactant could increase the optimum solubilization ratio from 21.5 to 25.0. However, the effect was insignificant enough to fall in the interpretation error range (Figure 2). The results of the phase behavior test confirmed the optimum ASP formulation: 1 wt % Na_2_CO_3_ as the alkali, 1 wt % 1:4 (*wt/wt*) LAS:DOSS mixture as the surfactant, and 5 wt % DGBE as the co-solvent. The phase distribution (the lower part of Figure 2) showed the region of Winsor type III that would be possibly present in the salinity range of 0.8 and 1.5 wt % NaCl. Winsor type I was expected during the preflush and the polymer drive processes, because the formation salinity was 0.6 wt % NaCl. The high salinity should be assigned to the ASP slug to generate type III.

Notably, DGBE (a glycol ether type) was more effective in increasing the optimum solubilization ratio than IBA (an alcohol type). The amounts of the surfactant and the alkali had insignificant influences on the solubilization ratios as well as the optimum salinity. The surfactant went to the micellar interface, whereas the co-solvent partitioned the oil and brine interface so that the co-solvent at the interface influenced efficiently the microemulsion phases [19]. DGBE had a higher molecular weight and the aqueous stability limits compared to IBA, which can yield a higher solubilization ratio [13,21].

**Figure 3:**
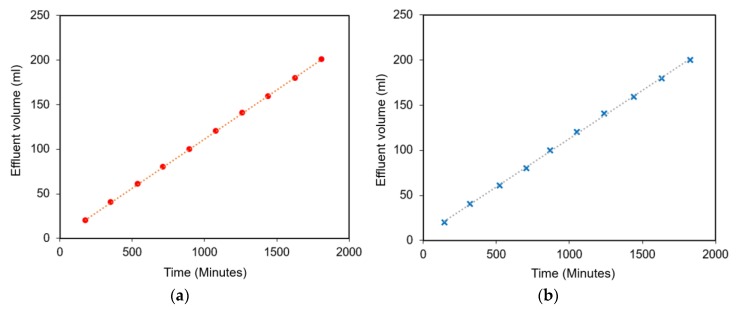
Plots of effluent volume as a function of time for hydrolyzed polyacrylamide (HPAM) polymer concentrations of 3000 mg/L (**a**) and 3300 mg/L (**b**) in the filtration ratio tests. The slope denotes the filtration ratio. It can be seen that a slop of 1.0 was obtained for the concentration of 3000 mg/L and a slop of 1.08 was obtained for the concentration of 3300 mg/L.

Figure 3 presents the FR and the slope of the observed points for the polymer concentrations, i.e., 3000 and 3300 mg/L. It can be seen that the effluent volume had a linear relationship with the time, demonstrating the slop of the plot, i.e., the FR, was constant. The FRs were 1.0 for a polymer concentration of 3000 mg/L and 1.08 for a polymer concentration of 3300 mg/L. Figure 3 proves that these concentrations had a lower risk of plugging at pore throats, i.e., the polymer hydration, since both were less than 1.2 (the criterion of FRs).

In summary, the optimum ASP formula can be 1 wt % Na_2_CO_3_ as the alkali, 1 wt % 1:4 (*wt/wt*) LAS:DOSS mixture as the surfactant, and 5 wt % DGBE as the co-solvent. The optimal design of ASP flooding required a high salinity of ASP slug with over 1.5 wt % NaCl considering the low salinity environment. The polymer concentrations should be 3000 or 3300 mg/L without any plugging at pore throats.

### 3.2. Coreflooding Tests

Coreflooding experiments were conducted to assess the field applicability of ASP formulas. The detailed designs of ASP flooding were as follows. The preflush using 0.6 wt % NaCl, which was used for the formation brine, was carried out, until no oil was recovered. The salinity of the ASP mixture was set as 1.5 wt % NaCl to make Winsor type III, since the residual brine reduced the ASP salinity but maintained it over 0.6 wt % NaCl. Two different amounts of ASP slug were examined, i.e., 0.37 pore volume (PV; a large volume of ASP slug) and 0.10 PV (a small volume of ASP slug). The polymer drive followed the ASP flooding with the 0.6 wt % NaCl salinity, and therefore its phase was type I. The polymer concentrations were 3000 mg/L in the ASP mixture and 3300 mg/L for the polymer drive. The sequence of phase types included in the preflush, the ASP mixture, and the polymer drive would be I–III–I.

**Figure 4:**
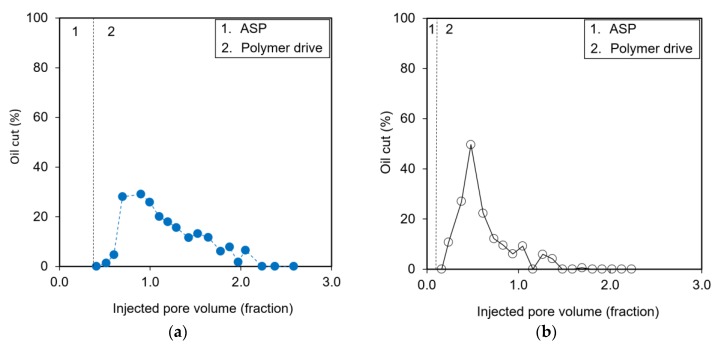
Performances of residual oil production (oil cut) implementing ASP flooding with different injection volumes of ASP slug: (**a**) an injected PV of 0.37 and (**b**) a PV of 0.10. The vertical dashed line represents the end of the ASP mixture injected into the core sample.

**Figure 5:**
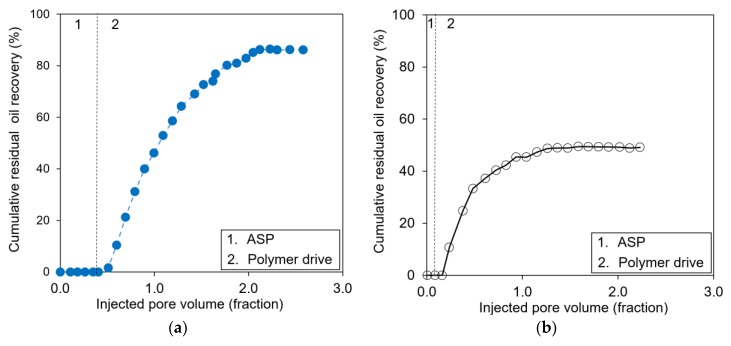
Cumulative residual oil recovery implementing ASP flooding with different injection volumes of ASP slug: (**a**) an injected PV of 0.37 and (**b**) an injected PV of 0.10. The vertical dashed line represents the end of the ASP mixture injected into the core sample.

Table 6 summarizes the detailed designs of ASP coreflooding tests and the results. Figure 4 demonstrates the oil cut, and Figure 5 describes the cumulative residual oil recovery with different ASP injection volumes, i.e., PV injection. A large amount of ASP slug was helpful in the production of more residual oil; the total oil recovery was 86.20% of the residual oil in the case of the 0.37 PV injection, while with the 0.10 PV injection the oil up to 48.96% was recovered. If the ASP mixture was injected into the core sample to generate a microemulsion and a lower interfacial tension, the residual oil could be recovered more. This study does not generate type II in the preflush, since the low-saline water flowed to demonstrate the field condition. It would be limited to compare the oil recovery in the case of type II–III–I. Large amounts of polymer displaced the oil bank (microemulsion) effectively to show the cumulative oil recovery rate no longer increased (Figure 5).

The results confirmed that the proposed ASP flooding system had positive synergy impacts on unrecovered oil after the waterflooding process. The first oil began at the end of the ASP injection, and the peak of the oil cut was observed in the region of the polymer drive. This performance meant that the ASP mixture mobilized the residual oil and turned it to a producible microemulsion. The polymer efficiently drove this mobilized oil. The lowest surfactant retention may be found in the type I microemulsion, since the surfactant was soluble in the water phase forming a water-external microemulsion, where the interfacial tension was high but easy to displace. On the other side, the type III microemulsion made the surfactant soluble in both oil and water, but the retention was higher than in the type I microemulsion. Thus, the coreflooding results showed that the high salinity of the ASP mixture generated a type III microemulsion and also a type I microemulsion created by the low salinity of the polymer drive would be positive under the low-salinity environment.

## 4. Conclusions

This paper suggested an optimal design of ASP flooding, i.e., ASP formulation, the salinity assignment for ASP components, and polymer concentrations, in order to recover the residual oil under the low-salinity environment. The optimal ASP formula consisted of 1 wt % Na_2_CO_3_ as the alkali, 1 wt% 1:4 (*wt*/*wt*) LAS:DOSS mixture as the surfactant, and 5 wt % DGBE as the co-solvent. The phase behavior test showed that the glycol-ether-type co-solvent with a large molecular weight could be effective and have a high aqueous stability limit on the optimum solubilization ratio. The type I–III–I sequence of phase types could recover the residual oil up to 86%, and the polymer drive with Winsor type I played a crucial role in recovering a three-phase microemulsion mobilized by the ASP mixture. The experimental results validated that the proposed design of ASP flooding would be effective in displacing the remaining oil that was not recoverable through waterflooding, despite the fact that low salinity limited the designs of salinity gradients and phase types.

## Figures and Tables

**Table 1:** Components of crude oil sampled at the target reservoir planned to conduct alkaline–surfactant–polymer (ASP) flooding.

Components	Concentration (mol %)	Weight Percentage (wt %)
Hydrogen sulfide (H_2_S)	0	0
Carbon dioxide (CO_2_)	0.3783	0.1044
Nitrogen (N_2_)	0.4645	0.0816
Methane (CH_4_)	11.2789	1.1348
Ethane (C_2_H_6_)	0.1622	0.0306
Propane (C_3_H_8_)	0.2503	0.0692
Isobutane (i-C_4_H_10_)	0.1832	0.0668
n-butane (n-C_4_H_10_)	0.4448	0.1621
Isopentane (i-C_5_H_12_)	2.3216	1.0505
n-pentane (n-C_5_H_12_)	2.6256	1.1881
Hexane (C_6_H_14_)	13.2666	7.1701
Heptane plus (+) (C_7_H_16_^+^)	68.6240	88.9418
Total	100	100

**Table 2:** Properties of available surfactants used in this work.

Surfactant	Trade Name (Abbreviation)	Active Matter (%)	Appearance
Dioctyl sulfosuccinate (C8–C8)	ASCODOSS (DOSS)	63.0–67.0	Clear liquid
Linear alkylbenzene sulfonate (C11–C13)	ASCO96 (LAS)	minimum: 96.0	Viscous amber liquid

**Table 3:** Components of crude oil sampled at the target reservoir planned to conduct ASP flooding.

Properties	Diethylene Glycol Monobutyl Ether (DGBE)	Isobutyl Alcohol (IBA)
Chemical formula	C_8_H_18_O_3_	C_4_H_10_O
Molar mass (g/mol)	162.229	74.122
Vapor pressure	-	9 mmHg (20 °C)
Density (g/cm^3^)	0.955	0.803
Melting point (°C)	−68	−108
Boiling point (°C)	231	108
Flash point (°C)	100	27
Appearance	Colorless clear liquid	Colorless liquid
Solubility in water	Soluble in water, ethanol, ethyl ether, and acetone	8.7 mL/100 mL

**Table 4:** Rock properties of Berea core samples used in coreflooding tests.

Sample	Diameter (cm)	Length (cm)	Porosity (%)	Permeability (md)	Pore Volume (PV; cm^3^)
Sample 1	3.80	14.8	19	200	31.86
Sample 2	3.79	14.2	17	180	27.83

**Table 5:** Results of phase behavior tests with different ASP formulations.

Case	ASP Formulation			Results		
	Alkali (wt % Na_2_CO_3_)	Surfactant (wt %, LAS:DOSS Ratio)	Co-solvent (wt %, Co-solvent)	Optimum salinity (wt % NaCl)	Optimum Solubilization Ratio (mL/mL)	Interfacial Tension (mN/m)
PB1	0	2 (1:2)	4, IBA	3.0	3.0	0.0333
PB2	0	2 (1:4)	4, IBA	2.0	2.5	0.0480
PB3	1	2 (1:4)	4, IBA	1.0	4.0	0.0188
PB4	1	1 (1:4)	2, IBA	1.0	5.0	0.0120
PB5	1	1 (1:4)	5, DGBE	1.25	21.5	6.49 × 10^−4^
PB6	1	0.9 (1:4)	5, DGBE	1.20	25.0	4.80 × 10^−4^

**Table 6:** Designs of the optimal ASP flooding and their residual oil recoveries.

Sample	Salinity (wt % NaCl)			PV Injection (ASP Mixture)	Residual Oil Recovery (%)
	Preflush	ASP Mixture	Polymer Drive		
Sample 1	0.6	1.5	0.6	0.37	86.20
Sample 2	0.6	1.5	0.6	0.10	48.96
